# Prevalence of ethanol and other potentially harmful excipients in pediatric oral medicines: survey of community pharmacies in a Nigerian City

**DOI:** 10.1186/s13104-019-4486-7

**Published:** 2019-07-26

**Authors:** Rebecca Soremekun, Irene Ogbuefi, Roseline Aderemi-Williams

**Affiliations:** 1 Department of Clinical Pharmacy, Faculty of Pharmacy, University of Lagos, Lagos, Nigeria; 2 Faculty of Public Health, West African Postgraduate College of Pharmacists, Lagos, Nigeria

**Keywords:** Pediatrics, Oral liquid medicines, Ethanol, Excipients, Community pharmacy

## Abstract

**Objective:**

Excipients are needed in the formulation of oral liquid medicines intended for children; they have however been reported to trigger safety issues. This study evaluated the concentrations and prevalence of ethanol and other potentially harmful excipients in pediatric formulations marketed in South Eastern Nigeria in line with international labeling guidelines and allowable daily limits (ADL). The study sampled oral pediatric formulations offered for sale in registered pharmacies. Those with accessible information leaflets were assessed for the presence and quantity of previously flagged excipients with potential to harm the pediatric population.

**Result:**

Of the 380 oral pediatric medicines, 140 provided access to list/quantity of ingredients. 47.9% (67) of the formulations contain at least one of the flagged excipients while the remaining only listed the active ingredients. Ethanol had the highest occurrence (62.7%) and was more in cough/cold medicines. A homeopathic cough and cold remedy had concentration of 90% v/v. Ethanol and sucrose in some formulations exhibited concentrations with a potential of crossing their approved daily intake (ADI) (1–90% v/v and 1.7 g–3.7 g/5 ml respectively). Ethanol use in studied pediatric formulations was quite high, with ethanol-containing formulations being prescribed for children 0–6 years and older. Only 26 (38.8%) completely satisfied the labelling requirements for ethanol containing formulations.

## Introduction

The pediatric population is a heterogeneous group ranging from newborns to adolescents with large pharmacokinetic and pharmacodynamic differences [1]. Children require particular oral dosage forms due to differences in swallowing abilities, taste preferences, and dosage requirements. There is a need therefore to adapt pediatric formulations such that their peculiar needs are met [2–4]. The production of these dosage forms is however froth with challenges which can only be overcome through the effective use of excipients. Excipients, though supposed to be inert, have shown the propensity to cause adverse reactions either alone or in drug-excipient reaction or even allergic reactions [5–8]. Two-thirds of newborns in 21 European countries who received common medicines were deemed to have been exposed to at least one potentially dangerous excipient in their medication, out of the eight examined [9]. Excipients are qualified by existing safety data with respect to the proposed level, duration of exposure or route of administration [10, 11].

A commonly used excipient is sugar and the American Heart Association has provided strong evidence supporting the association of added sugars with increased cardiovascular disease risk in children [12]. Ethanol is another excipient viewed with great concern by regulatory agencies. Maximum permissible limits have been set by international agencies in pediatric formulations, and a push for alcohol free medicines where possible is on in some countries [13]. Alcohol has a high permeability for the blood–brain barrier and Central Nervous System (CNS) effects have been reported [14].

This study sought to examine the concentration of alcohol and other previously flagged excipients in pediatric oral liquid formulations available for sale in Nigeria. Medicines are mostly obtained in community pharmacies on an out-of-pocket basis. The appropriateness of instructions contained in the medicine information leaflet was also evaluated.

## Main text

### Method

This study was carried out in Enugu, in the South Eastern part of Nigeria. The registered community pharmacy outlets in the state are 95 and 88% of them, situated in Enugu Urban area [15]. Children in the age bracket 0–14 years living in Enugu are put at 1,163,114 [16]. The study was a descriptive, quantitative study conducted over a 2-month period to examine oral pediatric formulations (offered for sale) for presence of excipients previously flagged in literature as having potential to harm the pediatric population [9]. These excipients are: ethanol, propylene glycol, sorbitol, saccharin sodium, aspartame, parabens, sodium benzoates, polysorbate 80 and the azo dyes.

Data was obtained from stock of pediatric oral formulations in pharmacies registered with the Pharmacist Council of Nigeria and with the presence of a pharmacist. Since stock of drugs in a particular region meeting the needs of same populations is likely to be predominantly of the same therapeutic category and from same suppliers, 10 pharmacies were purposively selected on the basis of stock strength and popularity of the store in the locality. The medicines were picked by direct selection of samples and relevant data obtained from the attached product leaflet using a semi-structured checklist, containing 18 items. The relevant variables were analyzed using appropriate descriptive and inferential statistics with the Statistical Package for Social Sciences (SPSS) Version 17 and presented as percentages, frequencies and summary statistics.

### Results

The sampled formulations covered a wide range of therapeutic groups: analgesics/antipyretics, antacids, anticonvulsant, antidiarrhoea, antihelmintics, antihistaminics, antimalarials, antimicrobials, cough/cold/flu medicines, and vitamin supplements. Of the 245 oral liquid medicines sampled, only 141 provided access to information on the ingredients of the formulation. These oral medicines fall into the following formulation groups: oral drops, three (2%), oral solution, six (4%), powder for reconstitution, 11 (8%), suspensions, 20 (14%) and syrups 101 (72%). Seventy-three (51%) of the 141 formulations did not disclose any information on excipients while the others represented excipients either as “excipients q.s.d” or “flavored syrup base q.s”. Fifty (69%) of the 73 inadequately labeled formulations were produced in Nigeria, 16 (22%) imported from India, three (4.1%) from other African countries, three (4.1%) from Europe and one (1.4%) from China.

The prevalence of excipients in the 68 formulations with available data on excipients studied is: 42 (63%) had one excipient, 14 (21%) contained two, six (9%) had three excipients, three (3%) contained four; two formulations (3%) had five while one (1.5%) liquid formulation contained 6 excipients. The compliance with ADI in the various formulations is presented in Table 1.

**Table 1:** Class of excipients in pediatric oral liquid medicines and compliance status

Class of excipients	Excipient	Frequency of use (n = 67)	Concentration in leaflet	Score rate^a^	ADI	Compliance status
Coloring agents/dyes	Tartrazine	1	Not stated	0/1	25 mg/kg	Cannot be determined
	Sunset yellow	8	Not stated	0/8	0.3 mg/kg	Cannot be determined
Preservatives	Methylparaben Proplyparaben	11 (As parabens)	0.045–0.18% 0.011–0.02%	3/11	5 mg/kg	Appropriate
	Sodium Benzoate	10	0.8% w/v	2/10	10 mg/kg	Appropriate
Solvents/co-solvents	Ethanol	42	1–90% v/v	26/42	5 g/day	Inappropriate
	Propylene Glycol	7	Not stated	0/7	10 mg/kg	Cannot be determined
Surfactants	Polysorbate 80	2	Not stated	0/2	10 mg/kg	Cannot be determined
Sweetners	Aspartame	2	0.03 g/5 ml	2/2	< 10 mg/kg	Appropriate
	Saccharin Sodium	6	5 mg/5 ml	1/6	2.5 mg/kg	Too high for infants
	Sorbitol	8	1 g–1.06 g/5 ml	2/8	0.3 mg/kg	Too high for infants and neonates
	Sucrose	11	1.7 g–3.7 g/5 ml	5/11	< 25 g/day	Too high for infants and neonates when added to sugar from foods

^a^Score rates = the number of formulations that stated the concentration of that excipient out of all the formulations containing the excipient e.g. 42 formulations contained ethanol but only 26 stated the concentration, so score rate for ethanol was 26/42

Ethanol was the excipient with the single highest usage in 42 (63%) oral medicines while sucrose a natural sweetener ranked second with 11 (16%) and combination of sweeteners in 27 (40%) formulations. Cough/Cold/Flu medicines topped the list of formulations with ethanol 23 (55%). Others are antihistaminics, 9%, antimicrobials, 7% and Vitamins/supplements, 17%. The level of compliance with recommended ADI in the use of ethanol in pediatric formulations is presented in Table 2. Seven (27%) formulations contained ethanol concentrations outside the stipulated permissible limit for children 0–6 years; four (15%) contained ethanol concentration outside the stipulated limits for children 6–12 years while five (19%) of the formulations contained concentrations greater than the stipulated amount for children 12 years and above.

**Table 2:** Compliance with recommendation status of ethanol in oral formulations

No of drugs indicating ethanol concentration	Concentration of ethanol stated	Approx. volume of alcohol in 5 ml dose	No that recommended formulation for age group					
			0–< 6 year		6–< 12 years		≥ 12 year	
			Yes	No	Yes	No	Yes	No
1	Up to 0.5%	0.025 ml	1	0	1	0	1	0
14	1–5%	0.05–0.25 ml	4	10	8	6	14	0
6	6–10%	0.3–0.5 ml	1	5	2	4	6	0
5	> 10%	> 0.5 ml	2	3	2	3	5	0
N = 26			7	18	4	13	5	0

No: Not recommended for use at the concentration in formulation; Yes: recommended for use at the concentration in formulation

### Discussion

Mandatory full labeling for all prescription and over-the-counter drugs had been recommended by the American Academy of Pediatrics [6, 17], whereas the Nigerian drug regulatory agency as at 2018, did not demand a full labelling of excipients, rather it required a full list of active ingredients and warning statements where appropriate. In 2006, better labelling compliance was reported in Brazil, with 72 drug inserts out of 73 giving details of all the excipients present in pediatric formulations studied [18].

A review of generic liquid drugs published in Brazil (2006 to 2011) revealed that 17.5% of these medicines contained ethanol in various concentrations [18]. Ethanol is used as a solvent, co-solvent, flavoring agent and preservative in liquid formulations and as an extracting solvent in herbal medicinal products [19]. In young children, ethanol may cause hypoglycemia and hypoglycemic seizures. This awareness of the potential hazards of ethanol as an excipient led to a push for a drop in the number of oral liquid medicines containing alcohol globally [20]. In 1989 there were 240 Food and Drug Administration (FDA) approved oral pharmaceuticals, listing ethanol as an excipient in the USA, an improvement from the 700 reported in 1978 [21]. The World Health Organization (WHO) proposed a limit for the ethanol content in OTC products to less than 0.5% for children less than 6 years old, less than 5% for children 6–12 years old and less than 10% for children over 12 years [22]. This WHO guideline is not been utilized in the regulation of oral liquid pediatric formulations in Nigeria. This study found pediatric formulations with ethanol concentrations greater than the recommended limits for all pediatric categories as presented in Table 2. In the management of fever for example; where medications are given every 8 h, a child under 6 years may consume up to 1.5 ml alcohol per day and if fever persists for at least 2 days, the child would have ingested 3 ml alcohol within the time frame. The high level of ethanol usage in Nigeria does not even take into consideration the possibility of accidental intake by children of all ages, the speed of metabolism and the distribution volume in children.

The French Medicines Agency had banned the use of ethanol in medicinal products intended for children unless absolutely necessary and if used, the amount should not produce blood concentration greater than 0.125 g/l. and the total volume of ethanol in the medicinal product should be adjusted so that a potentially lethal dose (3 g/kg) cannot be reached in the event of accidental poisoning in children [23].

Only 26 (61%), of the 42 formulations containing ethanol in this study stated the concentration used which ranged between 1 and 90% v/v. In a survey in New Zealand, 47 pediatric liquid medicines containing ethanol were found and 74% of these medications stated ethanol concentrations in the range of 0.6–76% v/v but the recommended dosing instructions are such that the ethanol consumed per dose was not expected to cause acute toxic effects [24]. Two homeopathic cough/cold formulations in this study used ethanol in concentrations of 65% v/v and 90% v/v as an extraction solvent. Herbal preparations are regarded as home remedies in Nigeria and are usually administered without a physician’s guidance, therefore self-dosing has the potential to expose children to chronic ethanol toxicity.

The concentration of sweeteners found in the pediatric medicines in this study ranged from 1.7 g to 3.7 g/5 ml. Four formulations had a combination of two sweeteners but only one of them (Azithromycin suspension) stated the concentrations of the combination of sweeteners (aspartame and sucrose), 0.03 g/5 ml and 3.7 g/5 mls respectively. Aspartame falls into the category of “Generally Regarded as Safe” excipients. It however has an ADI limit of 10 mg/kg beyond which it has the propensity to cause urticaria, angioedema, granulomatous panniculitis and cross-reactivity with sulfonamides [25]. Sugars are commonly used in pediatric medicines to improve palatability and hence acceptability. However there has been concern about their safety in this population. Eleven formulations indicated presence of sugar in the formulations studied while only five stated their sugar concentrations which ranged from 1.7 g to 3.7 g/5 ml. An assessment of most sold and/or prescribed liquid oral medicines for children in Southern Brazil revealed that 71% contained sugar and only 50.0% of the total medicines showed this information in their leaflet [26]. The American heart association recommends avoidance of added sugars for children < 2 years of age [12]. A 2 year old on a sugar-sweetened oral medication with concentration as stated in the Nigerian formulations runs the risk of consuming this much sugar in addition to sugar that will be ingested from other foods. Greater regulation is required for sugar content of pediatric medicines.

The azo dyes have generally been associated with hypersensitivity reactions in children but the formulations in this study containing the azo dyes, tartrazine and sunset yellow were completely silent on their concentrations (Table 1). The preservatives, methyl paraben and propyl paraben are applied in combinations in oral pharmaceutical formulations at concentrations ranging from 0.015 to 0.2% for methyl paraben and 0.02–0.06% for propyl paraben [27]. Only three (27%) of the 11 formulations containing parabens specified the concentrations used and they were all within limits. Infants are most often exposed to parabens because most oral drug formulations would require preservation. The concentration of sodium benzoate used as a preservative was stated in two of the 10 formulations containing sodium benzoate as 0.08% w/v. Neonates appear to lack the capacity to conjugate benzoates with glycine leading to the buildup of benzoic acid which can cause metabolic acidosis and neurotoxicity [27].

In the general assessment of the labelling of the pediatric products, the yardstick for certifying the labelling requirements complete was the presence of the product literature with the appropriate warning instructions clearly and explicitly written in English language. A childproof closure is recommended for medicinal products with ethanol content greater than 5% v/v [22]. Formulations with ethanol concentration greater than 100 mg/dose and above were in addition expected to state the ethanol equivalent of wine/beer per dose of the medication. Warning statements were clearly stated in 57% of the formulations while concentrations in mg/dose were stipulated in 19 (45%). A high compliance rate was observed for 15 (83) formulations which contained the statement indicating the ethanol equivalent of wine/beer in the formulations (Table 3).

**Table 3:** Labeling status in oral liquid preparations containing ethanol

No of formulations	Details required	Yes (%)
42	Leaflet insert present and legible	29 (69)
42	Warning statement included	24 (57)
42	Formulation has protective screw cap	29 (69)
42	Amount in mg per dose stated	19 (45)
18	Equiv. of wine/beer for 100 mg/dose and above stated	15 (83)

### Conclusion

Some manufacturers of pediatric oral liquid formulations sold in Nigeria considered the excipients used in their formulation as ‘trade secrets’ and therefore neither declared the names nor concentration on the labels and inserts. Ethanol with its attendant health hazards in children is a commonly used excipient in the pediatric formulations, with concentrations higher than stipulated in international guidelines and inappropriate labelling on the inserts. Protective screw caps, warning instructions, concentration in mg per dose were absent. Nigerian children exposed to these medications are therefore at risk of potential harm from excessive alcohol ingestion. There is a need for the regulatory agency in Nigeria to review labeling requirements for pediatric medications.

## Limitations

The findings of this study is limited to data obtained from pharmacies in the city of Enugu and the results may not be sufficient to make a general inference on pediatric medicines in circulation in Nigerian, though medicine suppliers in the country generally have a national reach.

## Data Availability

Data on the work can be found in the Fellowship project submitted to the West African Postgraduate College of Pharmacists and available from the corresponding author on reasonable request.

## Abbreviations

ADL: allowable daily limits
ADI: approved daily intake
CNS: Central Nervous System
FDA: Food and Drug Administration
WHO: World Health Organization
SPSS: Statistical Package for Social Sciences
